# Mechanism study on a plague outbreak driven by the construction of a large reservoir in southwest china (surveillance from 2000-2015)

**DOI:** 10.1371/journal.pntd.0005425

**Published:** 2017-03-03

**Authors:** Xin Wang, Xiaoyu Wei, Zhizhong Song, Mingliu Wang, Jinxiao Xi, Junrong Liang, Yun Liang, Ran Duan, Kecheng Tian, Yong Zhao, Guangpeng Tang, Lv You, Guirong Yang, Xuebin Liu, Yuhuang Chen, Jun Zeng, Shengrong Wu, Shoujun Luo, Gang Qin, Huijing Hao, Huaiqi Jing

**Affiliations:** 1 National Institute for Communicable Disease Control and Prevention, Chinese Center for Disease Control and Prevention, State Key Laboratory of Infectious Disease Prevention and Control, Collaborative Innovation Center for Diagnosis and Treatment of Infectious Diseases, Beijing, China; 2 Guizhou Provincial Center for Disease Control and Prevention, Guiyang, China; 3 Yunnan Institute for Endemic Diseases Control and Prevention, Dali, China; 4 Guangxi Zhuang Autonomous Region Center for Disease Control and Prevention, Nanning, China; 5 Gansu Provincial Center for Disease Control and Prevention, Lanzhou, China; 6 Xingyi Municipal Center for Disease Control and Prevention, Xingyi, China; 7 Luoping County Center for Disease Control and Prevention, Qujing, China; 8 Longlin Autonomous County Center for Disease Control and Prevention, Baise, China; 9 Xilin County Center for Disease Control and Prevention, Baise, China; University of California San Diego School of Medicine, UNITED STATES

## Abstract

**Background:**

Plague, a *Yersinia pestis* infection, is a fatal disease with tremendous transmission capacity. However, the mechanism of how the pathogen stays in a reservoir, circulates and then re-emerges is an enigma.

**Methodology/Principal findings:**

We studied a plague outbreak caused by the construction of a large reservoir in southwest China followed 16-years’ surveillance.

**Conclusions/Significance:**

The results show the prevalence of plague within the natural plague focus is closely related to the stability of local ecology. Before and during the decade of construction the reservoir on the Nanpan River, no confirmed plague has ever emerged. With the impoundment of reservoir and destruction of drowned farmland and vegetation, the infected rodent population previously dispersed was concentrated together in a flood-free area and turned a rest focus alive. Human plague broke out after the enzootic plague via the flea bite. With the construction completed and ecology gradually of human residential environment, animal population and type of vegetation settling down to a new balance, the natural plague foci returned to a rest period. With the rodent density decreased as some of them died, the flea density increased as the rodents lived near or in local farm houses where had more domestic animals, and human has a more concentrated population. In contrast, in the *Himalayan marmot* foci of the Qinghai-Tibet Plateau in the Qilian Mountains. There are few human inhabitants and the local ecology is relatively stable; plague is prevalence, showing no rest period. Thus the plague can be significantly affected by ecological shifts.

## Author summary

Plague in China is shown to be in 12 kinds of natural foci with a large scale geographic and complex structure. Each plague focus has a unique ecological environment, specific geographic regions, landscape characteristics and specific hosts and vectors for the maintenance and transmission of *Y*. *pestis*. Once the environment alters, the amount and density of hosts and vectors change and so does the survival of *Y*. *pestis*. Consequently, a rest or slightly active foci can com alive, rapidly cause plague among animals and/or humans and then disappear for long periods of time. A plague outbreak among animals and humans caused by the construction of a large reservoir in southwest China is reported in this study. The reservoir was located on the border between Yunnan-Guizhou Plateau and the Guangxi Hills on the Nanpan River, and the reservoir was not previously believed to contain the plague.

## Introduction

Plague, a *Yersinia pestis* infection, is a fatal disease with tremendous transmission capacity. However, the mechanism of how the pathogen stays in a reservoir, circulates and then re-emerges is an enigma [1]. Plague in China is shown to be in 12 regions of natural foci with a large scale geographic and complex structure [2]. Each plague focus has a unique ecological environment, specific geographic region, landscape characteristics, and specific hosts and vectors for the maintenance and transmission of *Y*. *pestis* [2–3]. The emergence of enzootic plague is a kind of natural reservoir for the pathogen [4]. Humans are infected with disease via a route such as bite by the flea from infected rodents [5–7]. *Y*. *pestis* continuously circulates between a host-vector-environment complex where the interaction determines the prevalence of the pathogen [5, 8–10]. Once the environment alters, the amount and density of hosts and vectors change and so does the survival of *Y*. *pestis* [11–12]. Consequently, a rest or slightly active foci can come alive, and rapidly cause plague among animals and or humans [13].

The plague outbreak took place along the repository of the Tianshengqiao reservoir in the border region of three provinces in southwest China: Xingyi County and Anlong County of Guizhou Province, Longlin County and Xilin County of Guangxi Province, and Luoping County of Yunnan Province. Since the outbreak, the region was identified as the *Rattus flavipectus* plague foci belonging to the mountainous area of Western Yunnan and the coastal area of Fujian and Guangxi. The focus has a subtropical monsoon humid climate and a residential farmland landscape (S1 Fig). The primary hosts are *Rattus flavipectus*, *Rattus norvegicus* and *Mus musculus*; and *Xenopsylla cheopis* and *Monopsyllus anisus* are the principal vectors. Tianshengqiao reservoir was built in 1987 and is 144 kilometers long, 181 meters high; and has a capacity of 10.3 billion cubic meters; water impoundment began from 1997 to 2000. The plague prevalent sites were located along reservoir banks and along new branches arising from the river impoundment. The residential areas were close to farmlands and rocky mountain, and often in poor sanitary conditions.

During the 10 years of reservoir construction, population density surged because construction workers relocated here. However, no enzootic or human plague was reported or infected dead rats found. When the impoundment inundation started in 1997, dead rats were present along the reservoir banks, and then by a few village banks in 1998. In 1999, the water capacity and drowned area increased, and the dead rats shot up and spread upstream from the reservoir banks to new branches. In 2000, dead rats were found in local farm houses in 15 foci along the banks (S1 Fig). In July of 2000, human plague occurred and it gradually declined until 2003 when no further cases were reported. An epidemiology study showed before the onset of plague, all the patients had dead rat contact and flea bite history. This outbreak of bubonic plague was transmitted by flea vector (*Xenopsylla cheopis*) and circulated between rat-flea- human.

## Methods

### Routine surveillance since the outbreak

Immediately after the outbreak and for 16 years plague surveillance was performed in Xingyi County of Guizhou Province, Longlin County and Xilin County of Guangxi Province and Luoping County of Yunnan Province. Enzootic plague was monitored at both fixed and mobile surveillance sites. Rodent hosts were measured for population structure, density and seasonal change; and importantly were examined for pathogen and serum detection of antibody and *Y*. *pestis* antigen. Human case reports were collected.

#### Rodent hosts, indicator animals and flea vectors

The cage-trap method was applied for estimation of rodent density (the number captured/the number of the rat cages) in the plague foci from surrounding farmhouses. Self-dead rodents (rodents that were found dead) from monitoring areas were collected. All the rats were classified and identified for population composition and the density was calculated from the captured rodents. Fleas carried by the rats were collected and counted; identification was performed under optical microscopy. They were pooled for *Y*. *pestis* culture. Dogs and cats infected *Y*. *pestis* by catching and feeding on infected rats, they have mild symptoms and are often self-healing, but specific F1-Antibody can be shown positive in the serum [14–16]. Dogs and cats are believed to be surveillance sentinels by measuring the antibody to F1 in the serum to predict plague outbreak risk [17–18]. Based on rodent surveillance, pathogen and sera were monitored in indicator animals, dogs and cats, to indirectly assess plague prevalence and they were sampled every year.

#### Pathogen detection

Samples from all the live and died rodents (including blood, liver, spleen, lung) were obtained for inoculating onto 2 selective agar plates (termed BIN agar, which is based on brain heart infusion agar with adding the selective agents of irgasan and cholate salts) for bacterial isolation at 28°C [19]. Flea were milled and inoculated onto culture medium. Biochemical tests such as arabinose, glycerine, rhamnose, and melibioseuse; Gram staining; bacteriophage lysis test; and specific polymerase chain reaction tests targeting F1 and *pla* genes, were used to identify the suspected isolates [19–20].

#### F1 antibody and antigen detection

Reverse indirect hemagglutination assay (RIHA) was used to detect the F1 antigen of *Y*. *pestis* from the tissues of rodents that were found dead [21]. The live rodents’ serum samples were collected from the femoral artery and were taken to detect antibody against F1 antigen by indirect hemagglutination assay (IHA). IHA was performed by including F1 antigen inhibition control and negative and positive controls. Antibody titers ≥1:20 (micro-plate method) was identified as positive; and required confirming analysis using the tube method according to the diagnosis criteria for plague [17, 22–25]. Captured rats that died of injury were not available for blood samples. Dogs and cats raised in Longlin County were tested for the F1 Antibody; they were calmed down and taken care of after venous blood collection; no pet animals died.

### Control surveillance

The natural plague foci in Gansu Province are relatively active, some of which are long prevalent, and some often alter between active and rest periods. We analyzed the surveillance and epidemiology data of some natural plague foci in Gansu from 1959 to 2014 and set the long active the *Himalayan marmot* foci of the Qilian-Altun Mountains as study control.

### Ethics statement

All the animals were handled according to the national criterion for animal plague investigation of China (Ethics Review Committee [Institutional Review Board (IRB)] of National Institute for Communicable Disease Control and Prevention, Chinese Center for Disease Control and Prevention) (License number: ICDC-2015001, ICDC-2015002) (S1 and S2 Files). The animals sample collection and detection protocols were carried out in accordance with relevant guidelines and regulations.

The human sample collection and detection protocols were carried out in accordance with relevant guidelines and regulations. All experimental procedures were approved by the Ethics Review Committee [Institutional Review Board (IRB)] of National Institute for Communicable Disease Control and Prevention, Chinese Center for Disease Control and Prevention (License number: ICDC-2015001, ICDC-2015002) (S1 and S2 Files). All adult subjects provided informed consent, and a parent or guardian of any child participant provided informed consent on their behalf. The informed consents were oral for all the participants, because the samples were too large; we couldn’t get all the written ones. The IRB approved the use of oral consent, and the information of consent contained the aim of the study, the usage of the patient’s samples, personal confidentiality agreement etc.

## Results

### Information on the plague outbreak

A total of 210 human cases were confirmed as bubonic plague with only one dead due to exacerbation to sepsis. Clinical specimens from patients including the bubo aspirates, bloody sputum, throat swabs, and necropsy organs (liver, lungs, spleen and heart) were used to isolate and identify *Y*. *pestis*. In 1999, plague first appeared in Xingyi County, Guizhou Province. Seventeen plague patients were first diagnosed as acute lymphadenitis. The IHA test results of them showed a high titer of specific antibody for *Y*. *pestis* (titer≥1:80); therefore these 17 patients were retrospectively confirmed as having plague infection.

**Fig 1:**
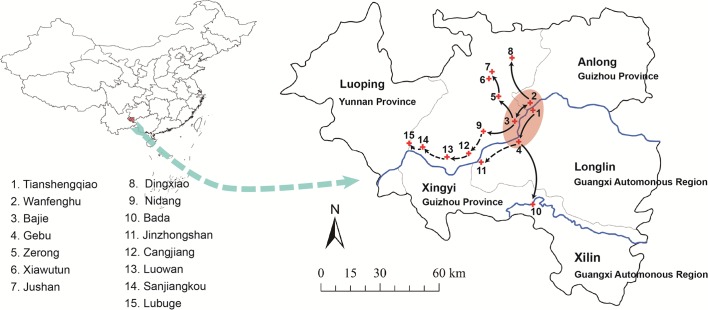
The spread of the plague outbreak 1–10: foci with both enzootic and human plague. 11–15: foci with only enzootic plague. Red region: the core focus of this outbreak; Solid arrows: enzootic and human plague spread path; Dotted arrow: enzootic but human plague spread path. The map is a Topographic Database of the National Fundamental Geographic Information System of China (NFGIS) which is shared and for free use, the URL link is http://www.tianditu.com/. The grey lines correspond to the delimited among different provinces.

The human plague outbreak peaked in 2000, the first site of the outbreak, Xingyi county, was most serious during this year. The circulation of plague extended from the core region to upstream in the reservoir and its branches to the center of Xingyi and to the county nearby the reservoir, Anlong and Longlin. Eighty-eight human plague cases were from Xingyi county, 44 from Bajie and 28 from Jushan, which were the most severe sites. Tianshengqiao in Longlin County, where the reservoir was, also experienced a serious human plague with 40 patients (Fig 1).

In 2001, with the adoption of prevention and control measures the plague extend from the core focus to upstream and to branches of the reservoir, with Xingyi (24 cases), Longlin (9 cases) and Xilin (5 cases).

In 2002, due to effective control measures, plague rapidly weakened. Jushan, at the end of a branch had the most patients (10 cases), while Nidang with six cases, Xiawutun with five cases, Bajie with two cases, and Zerong with one case (Table 1).

**Table 1:** The geographical and time distribution of human plague.

Foci	Year				
	1999*	2000	2001	2002	2003
Tianshengqiao	0	40	0	0	0
Wanfenghu	0	1	0	0	0
Bajie	13	44	1	2	1
Gebu	0	2	9	0	0
Zerong	4	1	2	1	0
Xiawutun	0	2	7	5	0
Jushan	0	28	1	10	0
Dingxiao	0	8	0	0	0
Nidang	0	4	13	6	0
Bada	0	0	5	0	0

* The seventeen patients in 1999 were first diagnosed as acute lymphadenitis. They were confirmed as plague patients using the IHA test after the outbreak in 2000.

In 2003, only one case appeared at Bajie in the core focus, Xingyi (Table 1 and Fig 1), and was the last case. The index and the last case both emerged in the most seriously impacted county, Xingyi.

#### Regional distribution of foci and spread path of plague

Before the water impoundment of the reservoir, a large number of residents lived along the banks of Nanpan River with large quantities of farmland. After filling, the river was wider, and the capacity increased; and as a consequence the farmland along the banks was submerged, covering a total area of 177.75 square kilometers; and brought about 42,050 immigrants (S1A, S1B and S1D Fig). Human plague started in the villages along river banks of Tianshengqiao Reservoir (Tianshengqiao, Wanfenghu, Bajie, Gebu were the core foci of the outbreak), and gradually spread upstream and to the branches of the reservoir. Most foci were distributed in villages along the banks and branches of the reservoir. Among them, Tianshengqiao, Wanfenghu, Bajie, Gebu, Zerong, Xiawutun, Jushan, Dingxiao, Nidang, and Bada were the sites where both human plague and enzootic plague occurred. In Jinzhongshan, Cangjiang, Luowan Sanjiangkou, and Lubuge, only enzootic plague took place and no human cases were reported; these places belonged to the subsequent dissemination region (Figs 1 and S1C). Dead rodents, from which *Y*.*pestis* were isolated, were found in farmhouses in Luoping, Yunnan Province located upstream of the reservoir and these farmhouses were only 6 kilometers away from the bank of the tailrace submerged by the reservoir. Further, a number of rodents were found dead in the field beside the farmhouses (S1E Fig).

### Routine surveillance

#### Surveillance of rodent density after the outbreak

A small amount of dead rats was found along the reservoir bank in 1998. The dead rats increased in 1999 with the impoundment where the submerged area increased. By 2000, dead rats were found not only along the reservoir banks, but also in nearby resident houses along the riverside. The human plague began in July of 2000, and prevalent in areas where the dead rats increased with the water impoundment processes, and was prevalent in the areas where increased dead rats were found.

The surveillance was conducted in Longlin, Xilin, Luoping and Xingyi Counties from 2000 to 2014. The average rodent capture rate from the four counties was 3.77% (30,524 /809,652). Total capture rate from low to high were respectively Xingyi 3.04% (6,751/221,820), Longlin 3.06% (7,796/254,655), Xilin 3.94% (8,855/224,850), and Luoping 6.57% (7,122/108,327). From 2000 to 2001 during the outbreak, Xingyi enforced de-ratting and thus failed to carry out normal rodent density surveillance. With transverse comparison of the rodent capture rate in 4 counties in 2002 and 2003, Longlin was 3.20% and 2.78%, Xingyi 6.26% and 4.49%, Xilin 11.50% and 5.26%, and Luoping 8.62% and 12.25%, respectively. The rodent composition indicated *R*. *flavipectus* was the dominant host in Xingyi, Xilin, and Longlin, while it was *R*. *norvegicus* in Luoping. We found respectively:

Xingyi: *R*. *flavipectus* (41.1%) > *R*. *norvegicus* (38.0%) > *M*. *musculus* (11.7%);

Xilin: *R*. *flavipectus* (85.3%) > *M*. *musculus* (10.0%) > *R*. *norvegicus* (11.7%);

Longlin: *R*. *flavipectus* (82.5%) > *M*. *musculus* (11.0%) > *R*. *rattus sladeni* (2.3%);

Luoping: *R*. *norvegicus* (57.4%) > *R*. *flavipectus* (32.5%) > *M*. *musculus* (8.0%).

#### *Y*. *pestis* isolation from rodents

**Fig 2:**
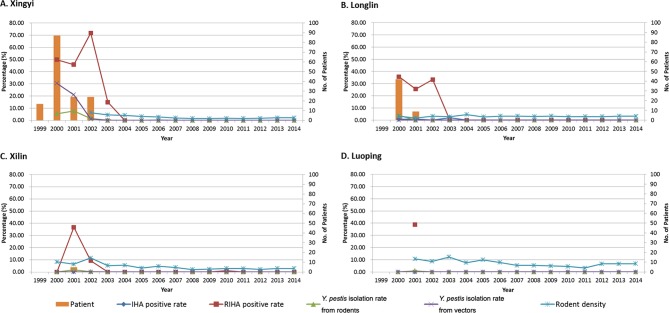
Human plague cases and surveillance of rodent pathogen and serology in Xingyi, Longlin, Luoping and Xilin from 1999 to 2014. The histogram means the number of the patients, with a total of 210 human cases were confirmed. The dark blue line means the percentage of IHA positive rate of rodents, a total of 22,472 rodent serum samples were collected and 12 were positive; the red line means the percentage of RIHA positive rate of rodents, a total of 1,047 rodent liver and spleen samples were collected and 297 were positive; the green line means the percentage of *Y*. *pestis* isolation rate from rodents, 64 of *Y*. *pestis* were isolated from 3,9166 rodents; the purple line means the percentage of the percentage of *Y*. *pestis* isolation rate from vectors, 12 of *Y*. *pestis* were isolated from 1,5696 vectors; the light blue line means the percentage of rodent capture rate, the average rodent capture rate from the four counties was 3.77% (30,524 /809,652).

Tissues from a total of 39,166 rodents were cultured from the four counties from 2000 to 2014. *Y*. *pestis* strains could only be isolated from 2000, 2001 and 2002 consistent with the emergence of human cases. A total of 64 strains were isolated where the average carriage rate was 0.16%; and respectively from high to low were Xingyi 0.21% (27/12,680), Longlin 0.16% (13/7,945), Xilin 0.16% (18/10,912), and Luoping 0.08% (6/7,629). Specifically, in Xingyi we isolated strains from 2000 to 2002: 5.45%, 7.69% and 1.55%. And from Xilin we isolated *Y pestis* in 2001 and 2002, 1.68% and 0.31% respectively. From Longlin, we only isolated strains in 2000 (3.82%). From Luoping strains were isolated in 2001 (0.92%) (Fig 2, Table 2). The results confirm *R*. *flavipectus* as the dominant rodent host in the plague foci. Host density investigations show *R*. *flavipectus* was the highest in all of the counties except Luoping, where *R*. *norvegicus* is slightly higher than *R*. *flavipectus*. However, *Y*. *pestis* strains were predominantly isolated from *R*. *flavipectus* in all counties.

**Table 2:** *Y*. *pestis* isolated from rodents *.

Year	Longlin					Luoping				Xilin				Xingyi				
	Rodents No.	Isolation rate (%)	Strains isolated from			Rodents No.	Isolation rate (%)	Strains isolated from		Rodents No.	Isolation rate (%)	Strains isolated from		Rodents No.	Isolation rate (%)	Strains isolated from		
			*R*.*f*	*R*.*n*	*M*.*m*			*R*. *f*	*R*. *n*			*R*. *f*	*R*. *n*			*R*. *f*	*R*. *n*	*R*. *r*
2000	340	3.82	12	0	1	356	0	0	0	676	0	0	0	275	5.45	15	0	0
2001	282	0	0	0	0	655	0.92	5	1	833	1.68	14	0	39	7.69	3	0	0
2002	318	0	0	0	0	592	0	0	0	1295	0.31	4	0	579	1.55	7	1	1
2003	341	0	0	0	0	494	0	0	0	772	0	0	0	1564	0	0	0	0
2004	607	0	0	0	0	765	0	0	0	669	0	0	0	1958	0	0	0	0
2005	298	0	0	0	0	618	0	0	0	883	0	0	0	1333	0	0	0	0
2006	534	0	0	0	0	632	0	0	0	921	0	0	0	854	0	0	0	0
2007	709	0	0	0	0	602	0	0	0	706	0	0	0	847	0	0	0	0
2008	648	0	0	0	0	410	0	0	0	563	0	0	0	816	0	0	0	0
2009	737	0	0	0	0	398	0	0	0	508	0	0	0	576	0	0	0	0
2010	606	0	0	0	0	402	0	0	0	667	0	0	0	769	0	0	0	0
2011	632	0	0	0	0	414	0	0	0	859	0	0	0	859	0	0	0	0
2012	614	0	0	0	0	401	0	0	0	575	0	0	0	788	0	0	0	0
2013	712	0	0	0	0	440	0	0	0	585	0	0	0	773	0	0	0	0
2014	567	0	0	0	0	450	0	0	0	400	0	0	0	650	0	0	0	0
Total	7945	0.16	12	0	1	7629	0.08	5	1	10912	0.16	18	0	12680	0.21	25	1	1

*)*R.f: R. flavipectus; R.n: R. norvegicus; M.m: M. musculus; R.r: R. rattus sladeni

#### The detection of F1 antibody

A total of 22,472 rodent serum samples were collected from the four counties during 2000–2014 and F1 antibody tests were performed using the indirect hemagglutination test (IHA). Twelve were positive, and the average positive rate was 0.053%. We collected 6,752 serum samples from Xingyi during 2002–2014 (routine surveillance was not performed from 2000 to 2001 due to the de-ratting operation), and five were positive with positive rates were respectively 1.58% (3/190) in 2002, 0.14% (1/700) in 2003, and 0.2% (1/500) in 2006. Among 4,810 samples collected in Longlin, six were positive (all *R*. *flavipectus*). Among 8,543 samples collected in Xilin, only one *R*. *flavipectus* was positive in 2001, and its positive rate was 0.15% (1/674). All 2,367 serum samples collected in Luoping during 2000–2014 were IHA negative (Fig 2, Table 3). IHA positive results primarily came from *R*. *flavipectus*, consistent with the strain isolation results. Besides positive samples detected during the plague epidemics, one positive serum came from *R*. *flavipectus* in Xingyi in the rest phase (in 2006).

**Table 3:** The detection of F1 antibody from rodents *.

Year	Longlin				Luopin		Xilin				Xingyi				
	No. of rodent	Positive No.	Positive rate	*R*. *f*	No. of rodents detected	Positive No.	No. of rodents detected	Positive No.	Positive rate	*R*. *f*	No. of rodents detected	Positive No.	Positive rate	*R*. *f*	*R*. *n*
2000	150	1	0.67	1	314	0	565	0	0.00	0	Non detected				
2001	125	1	0.80	1	219	0	674	1	0.15	1					
2002	230	0	0.00	0	206	0	1144	0	0.00	0	190	3	1.58	3	0
2003	194	4	2.06	4	203	0	531	0	0.00	0	700	1	0.14	0	1
2004	457	0	0.00	0	126	0	460	0	0.00	0	685	0	0.00	0	0
2005	218	0	0.00	0	190	0	555	0	0.00	0	481	0	0.00	0	0
2006	353	0	0.00	0	132	0	682	0	0.00	0	500	1	0.20	0	1
2007	409	0	0.00	0	101	0	512	0	0.00	0	501	0	0.00	0	0
2008	342	0	0.00	0	114	0	532	0	0.00	0	518	0	0.00	0	0
2009	396	0	0.00	0	112	0	397	0	0.00	0	554	0	0.00	0	0
2010	393	0	0.00	0	104	0	510	0	0.00	0	507	0	0.00	0	0
2011	388	0	0.00	0	117	0	514	0	0.00	0	555	0	0.00	0	0
2012	368	0	0.00	0	104	0	534	0	0.00	0	544	0	0.00	0	0
2013	435	0	0.00	0	174	0	535	0	0.00	0	545	0	0.00	0	0
2014	352	0	0.00	0	151	0	398	0	0.00	0	472	0	0.00	0	0
Total	4810	6	0.12	6	2367	0	8543	1	0.01	1	6752	5	0.07	3	2

*)*R.f: R. flavipectu; R.n: R. norvegicus

#### F1 antigen data from liver and spleen samples from naturally dead rodents

Liver and spleen samples, 1,047 in total, collected from naturally dead rodents in the four counties during 2000–2014, were tested using the reverse indirect hemagglutination test (RIHA). The average positive rate was 25.21%, and positive specimens primarily appeared in 2000–2003; and generally in accordance with the emergence of the human cases. Positive rates from high to low were respectively Xingyi 45.77% (195/426), Luoping 38.89% (21/54), Longlin 16.13% (25/155), and Xilin 5.58% (23/412). The positive results in Xingyi County gathered in 2000–2003 were respectively, 49.82%, 45.76%, 71.79%, and 15.00%, while the rest of the years are negative. The positive samples of Longlin gathered in 2000–2002 were, respectively, 35.71%, 25.49% and 33.33%, while the rest negative. Xilin was 36.73% and 9.30% in 2001 and 2002 respectively, and in 2010, during the rest period, one *R*. *flavipectus* sample tested positive. The RIHA test in Luoping County was performed only in 2001, and its positive rate was 38.89% (Fig 2).

#### Surveillance of indicator animals (high-resistant animals)

**Fig 3:**
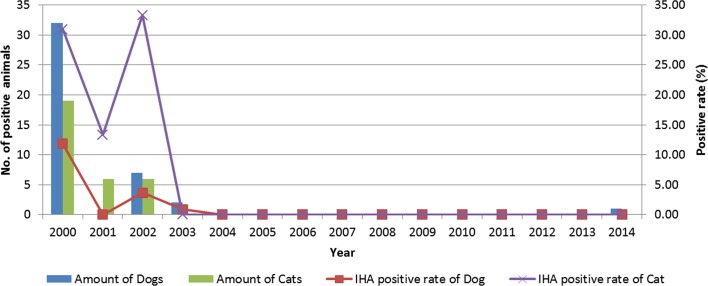
The IHA surveillance of indicator animals in natural foci from 2000 to 2014. The y axis "No. of positive animals" on the left side correspond to the histogram and "positive rate" on the right side correspond to the line chart. A total of 2,054 dogs were tested with 28 were positive; 59 cats were tested with 10 were positive.

Dogs and cats are of important indicator animals to predict the epidemic intensity of plague. Both China and other countries have related reports that these two indicator animals have low sensitivity and a high tolerance for *Y*. *pestis* [15–17]. The serological surveillance number of indicator animals between plague foci and non-plague foci in Longlin County from 2000 to 2014 were 2,113 and 1,592 respectively, the result was as follows: in the plague foci, the IHA positive rate from cats during 2000–2002 were 30.95%, 13.33% and 33.33% respectively, and in the rest of the years were negative; the IHA positive rate from dogs from 2000, 2002 and 2003 were 11.85%, 3.66% and 0.91%, respectively, and the rest of the years negative. In contrast, the results from dogs and cats in non-plague foci were all negative. Cats have a special addiction to small rodents, thus the cats' positive rate was higher than farm dogs (Fig 3).

#### Flea vector surveillance

During 2000–2014, 15,696 vectors were cultured for *Y*. *pestis* isolation in the four counties, among which only Xingyi County, the severest human plague foci, had isolated strains. The time distribution of positive vectors was coincident with human case appearance. In addition, 6,019 vectors were collected in Longlin, 3,898 in Luoping, and 2,537 in Xilin, all of which were negative for *Y*. *pestis* strains (Fig 2).

We collected the rodents including live rats and dead rats, plague bacteria could be separated from the dead bodies of rats, and however serum samples of dead rats were not collected. Therefore, the IHA test wasn’t be performed. The plague bacteria were isolated from the majority of IHA positive rodents in our study. Some serum samples were positive for IHA test, while couldn’t isolated the bacteria.

### Surveillance for control foci

#### The epidemic situation in a long-active natural plague foci

**Fig 4:**
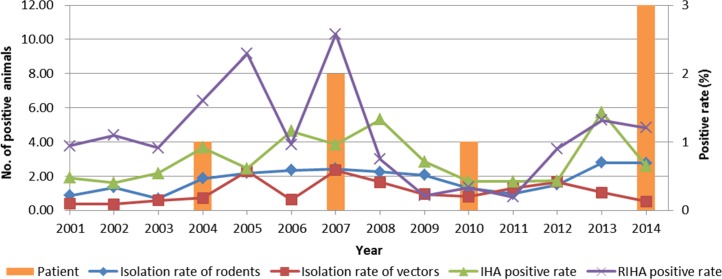
Active surveillance and epidemic record in the plague natural focus of *Himalayan marmot* in Gansu Province from 2001 to 2014. The y axis "No. of positive animals" on the left side correspond to the histogram and "positive rate" on the right side correspond to the line chart. The histogram means the number of the patients, with a total of 7 human cases were confirmed. The green line means the percentage of IHA positive rate of rodents, a total of 19,838 rodent serum samples were collected and 601 were positive; the purple line means the percentage of RIHA positive rate of rodents, a total of 2,047 rodent liver and spleen samples were collected and 89 were positive; the blue line means the percentage of *Y*. *pestis* isolation rate from rodents, 458 of *Y*. *pestis* were isolated from 25,005 rodents; the red line means the percentage of the percentage of *Y*. *pestis* isolation rate from vectors, 166 of *Y*. *pestis* were isolated from 1,5195 vectors.

In Gansu Province from 1959 to 2014, outbreaks of human plague happened 30 times, in which 70 patients were diagnosed; and 43 of them died with a fatality rate of 61.43%. Between 2001 and 2014, the *Himalayan marmot* foci of Qilian Mountain was particularly active, with one human case in 2004, two cases in 2007, one case in 2010, and three cases in 2014. This focus belongs to the China *Himalayan marmot* foci on the Qinghai-Tibet Plateau, where the plague is enzootic among animals. The surveillance results during 2001–2014 showed that the isolation rate of host was 1.83%; the isolation rate of vectors was 1.09%; the average IHA positive rate was 3.03%; and the average RIHA positive rate was 4.35% (Fig 4).

#### An epidemic in a natural foci turning into the rest phase after an active phase

The Gannan Tibetan Autonomous Prefecture is another natural focus in Gansu Province, located on the *Marmatahimalayana* plague focus of Qinghai-Tibet plateau. The first human plague broke out in 1958, and later the plague was enzootic between animals in the local grassland habitat of *Marmatahimalayana* until 1970. During 1971 to 1990, no plague was detected in this region. But in 1991, two marmots were detected F1 Ab positive, and then five in 1998, two in 1999, and one in 2000; the highest titer was 1:160. In 1999 a large number of marmots died. Three strains of *Y*. *pestis* were isolated from *Marmatahimalayana*, and eight samples were positive for RIHA (five at 1:100, and three at 1:200).

## Discussion

Plague has a strict geographical distribution, which is called plague natural foci. Plague invades, fades out, and re-invades alternately existing in animals where some plague natural foci seem to disappear permanently while others re-invades after a rest period of many years. The reasons for plague's persistence, abrupt disappearance in natural environment are poorly understood [26–27].

Here we report an outbreak of plague occurring on the border between Guizhou, Guangxi and Yunnan resulting from the building of a large reservoir. The last disease related to the death in rats, based on historical records in Luoping County, Yunnan Province, occurred from 1882 to 1892. The pandemic spread over eleven years, leaving a human death toll of ten thousand. Although it has not been confirmed today, we speculated it was a plague through the description of the event and the characteristics of the outbreak. Since then no similar incident has been reported in the area for a hundred years. Until 1997, the animal and human plague re-invaded after the impoundment of reservoir in the late 1990s. The plague lasted until 2004, when human plague disappeared and animal plague faded out with only a few rodents carrying the F1 antibody or antigen. We speculate the valley region along the Nanpan River may be a natural plague focus previously in history where under certain conditions, the plague invaded and faded out into a long rest period. The *Y*. *pestis* bacteria circulated in a very limited range and formed several micro-foci with discrete distribution and were hard to discover as the infection was in a long resting stage. When the dam began to fill the valley, the ecological environment experienced a violent change in a short period of time with the water level increasing. Animal migration resulted in the population density and a distribution change in rodent hosts. The plague-infected rodents previously in scattered distributed micro-focus gathered; then, the plague re-invaded. When the animals adapted to the new habitats and recovered to a stable state, the plague foci returned to the resting stage. This highly supports the inner preservation theory of plague [20], which states *Y*. *pestis* continuously exists within plague foci in the resting stage; however it is weakly prevalent and difficult to detect.

A small amount of dead rats were found along the reservoir bank in 1998. The dead rats increased in 1999 with the impoundment where the submerged area increased. By 2000, dead rats were found not only along the reservoir banks, but also in nearby resident houses along the riverside. The plague was prevalent among the rodents after with a large number of self-dead rodents were found in the epidemic area. The human plague began in July of 2000, and prevalent in areas where the dead rats increased with the water impoundment processes, no dead rats no plague cases. Plague initially existed in the first drown areas by the impoundment of the reservoir (Fig 1, the red zone), foci were mainly distributed in the submerged regions along the riverside and its branches, later spreading towards the upstream and to branches of the reservoir (Figs 1 and S1 show the transmission route). During the ten years constructing the reservoir, there were no epidemic plague among human and animals.

Enzootic and human plague has persisted until 2003, although the initial confirmed case was in 2000, the first cases can be traced back to 1999 when 25 patients had acute lymphadenitis according to the local hospital records. After the outbreak in 2000, they were tested with IHA, and 17 of 25 were detected positive with F1 antibodies (with titers>1:80), which were then diagnosed as plague cases. This fully confirms plague cases occurred in 1999 without being discovered, and the dead rodents existing in the early stage of impoundment were associated with the plague infection. A total of 210 human cases occurred in this outbreak and were all confirmed as bubonic plague with only one dead because the patient delayed in seeing a doctor with exacerbation to sepsis.

Plague spread requires both a high abundance of hosts and a sufficient number of active fleas. The surveillance results show the plague epidemic was worst in Xingyi County, however the rodents captured by cage traps was lower than Xilin and Luoping County, only higher than Longlin County, which may have been due to the de-ratting before the monitoring and a large number of rodent death during the plague outbreak. However, the *Y*. *pestis* isolation rate in rodents in Xingyi was much higher than the other three counties from 2000 to 2002. In this study, *Y*. *pestis* was only detected from the fleas [7, 28] in the core outbreak zone, Xingyi County, and the bacteria isolation rate from fleas was in accordance with the number of plague cases among people from 2000 to 2002. After the plague epidemic (after 2003), plague retreated and the natural foci seemed to rest for a long time, and it was difficult to detect in animals in routine surveillance from 2004 to 2014 (Fig 2). Only one IHA positive *R*. *flavipectus* was discovered in Xingyi County in 2006 (positive rate 0.2%), and one RIHA positive naturally dead *R*. *flavipectus* in Xilin County in 2010 (positive rate 1.08%). Thus, the plague has retreated over the past decade, but it has not disappeared. It was difficult to detect the extremely low levels of plague prevalence in the resting stage due to the limited number of naturally dying animals available during the conventional monitoring. To find a better way of monitoring, it was necessary to strengthen the routine surveillance.

Though cats could be infected by *Y*. *pestis*, they are believed to be a surveillance sentinel by measuring the antibody to F1 in the serum to predict plague outbreak risk [17–18]. The cats can resistant to *Y*. *Pestis* infection [16] and was a mediator to facilitate transfer of fleas as a source of bubonic or septicemic plague [29]. As we all know China is one of the most prevalent countries of plague, and therefore the responsibility of the government was to define each plague natural focus so the people can understand the major characteristics of plague and be aware of its dangers. However, the region in this study was not defined as a potential plague natural focus before 2000, so the government did not pay close attention and take emergency measures until the outbreak of plague cases. It suggested that timely recognition and judgment of the natural foci of plague is vital in the treatment and prevention of this acute and highly infectious disease. It was easy to overlook the dangers of plague especially for the resting plague natural foci where it is difficult to discover animal plague at a low epidemic stage. The plague will re-invade when the environment or climate change, and threats of outbreaks may thus be increased where local humans live in close contact with rodents and fleas (or other wildlife) harboring endemic plague.

Emergence, spread, persistence and fade-out of plague can alternate for varying lengths of time in the plague natural foci [13], the reasons for plague’s persistence and abrupt disappearance are poorly understood. We speculate the plague occurs in a particular habitat, the micro-focus, and *Y*. *pestis* is preserved in this region during the resting stage. Similar to our study, the human plague broke out in *Spermophilus dauricus alashanicus* plague natural foci in Huining County, Gansu Province in 1962, and a large number of rodents died before the epidemic. In the Gannan Tibetan Autonomous Prefecture, Gansu Province, located on the Qinghai-Tibet plateau plague focus of *Marmatahimalayana*, human plague started in 1958 and the epizootic plague persisted among marmot until 1970. The Qilian Mountain region of Gansu Province which is also located on the Qinghai-Tibet plateau plague focus of *Marmatahimalayana* is one of the most active natural plague foci in China [15, 30], witnessed by continued outbreaks of plague in animals year after year with *Y*. *pestis* persistently isolated from marmots (except from 1979 to 1981); especially in 2014, three human plague patients were found within three months.

There is a distinct difference in the ecology environment, human population and animal density in these three areas. Huining County with a high concentration of people (580,200 people on 5,657 km^2^). After the outbreak of plague in the 1960s, the local people cultivated the virgin land, changed the landscape to destroy the suitable environment for *Spermophilus dauricus alashanicus*. As a result, the rodent densities were decreased, and the plague prevalence faded. The Gannan Tibetan Autonomous Prefecture was similar with a large population and tourism development, so the active and rest period of plague alternate in this region. However, sparsely populated in Subei (12,000 people on 55,264 km^2^) and Akesai County (8,000 people on 29,142 km^2^) in the Qilianshan region were beneficial to the persistence of plague. Fewer construction projects and dispersed residences were also conducive to keeping the ecological balance of plague, with sporadic or only local breakouts (Fig 4) and few human cases occurred, and it is common for re-emerging rodent-derived plague epidemics after decades in many regions of the world [11, 13]. For instance, in Kazakhstan, the bacterium *Y*. *pestis* circulates in natural populations of gerbils. Analysis of field data collected between 1955 and 1996 shows that plague invades, fades out, and re-invades in response to fluctuations in the abundance of its main reservoir host, the great gerbil (*Rhombomys opimus*) [13].

The spread of plague requires interactions with the pathogen, its ecology, hosts and vectors [12–13, 31]. Here we show a typical example of the re-emergence of plague driven by the construction of a large reservoir. Through the comparison study of the Qinghai-Tibet plateau plague focus of *Marmatahimalayana* in Gansu province, we confirmed that the bacterium *Y*. *pestis* can long-term persist in the natural plague focus [27, 32] where it exists within the micro-focus in the rest stage, and persists covertly among its hosts and vectors. When the host density increases or abiotic factors change, a local outbreak of plague occurs. The inner preservation theory of plague is supported by this outbreak and the subsequent 16-year continuous monitoring, states that *Y*. *pestis* is inherent within a natural resting plague focus.

## Supporting information

S1 Checklist STROBE Checklist. (DOC)

S1 Fig Landscape and environment change due to the impoundment of the Tianshengqiao Reservoir, and the spread path of the plague. A: Landscape before the impoundment; the black dots represent foci with both enzootic and human plague cases; B: Landscape after the impoundment; the yellow dots represent foci with both enzootic and human plague cases; the red dots represent foci only with enzootic plague but no human plague; C: The enlarged view of black rectangle of B; the yellow dots represent the foci with both enzootic and human plague cases; yellow arrows represent the spread path of enzootic and human plague; the red dots represent the foci with only enzootic plague but no human plague; red arrows represent the subsequent spread path of enzootic plague. D: Aerial photo of the Tianshengqiao reservoir upstream and downstream. E: The farmhouse on the bank of the tailrace where dead rodents were found and *Y*. *pestis* strains were isolated; the yellow box indicated the farmhouses on the banks. F: Environment of tailrace banks. The map is a Topographic Database of the National Fundamental Geographic Information System of China (NFGIS) which is shared and for free use, the URL link was http://www.tianditu.com/.
(TIF)

S1 File The copy of ethical approval documents of funded program 2. (PDF)

S2 File The copy of ethical approval documents of funded program 3. (PDF)
