# Supplementary material for: Mechanism study on a plague outbreak driven by the construction of a large reservoir in southwest china (surveillance from 2000-2015)
Source: PLoS Negl Trop Dis. 2017 Mar 3;11(3):e0005425. doi: 10.1371/journal.pntd.0005425 (PMC5352140; doi:10.1371/journal.pntd.0005425)
Supplement: S1 Checklist — (DOC) [file pntd.0005425.s001.doc]

STROBE Statement—checklist of items that should be included in reports of observational studies

|  | Item No | Recommendation |
| --- | --- | --- |
| **Title and abstract** | 1 | Mechanism study on a plague outbreak driven by the construction of a large reservoir in southwest China (surveillance from 2000-2015) |
|  |  | **Background**  Plague, a *Yersinia pestis* infection, is a fatal disease with tremendous transmission capacity. However, the mechanism of how the pathogen stays in a reservoir, circulates and then re-emerges is an enigma.  **Methodology/Principal findings**  We studied a plague outbreak caused by the construction of a large reservoir in southwest China followed 16-years’ surveillance.  **Conclusions/Significance**  The results show the prevalence of plague within the natural plague focus is closely related to the stability of local ecology. Before and during the decade of construction the reservoir on the Nanpan River, no confirmed plague has ever emerged. With the impoundment of reservoir and destroy of drowned farmland and vegetation, the infected rodent population previously dispersed was concentrated together to flood-free area and turned a rest focus alive. Human plague broke out after the enzootic plague via the flea bite. With the construction completed and ecology gradually of human residential environment, animal population and type of vegetation settling down to a new balance, the natural plague foci returned to a rest period, with the rodent density decreased as some of them died, the flea density increased as the rodent lived near or in local farm houses where had more domestic animals, and human has a more concentrated population.  In contrast, in the Himalayan marmot foci of the Qinghai-Tibet Plateau in the Qilian Mountains. There are few human inhabitants and the local ecology is relatively stable; plague is prevalence, showing no rest period. Thus, the plague can significant affects by the massive changes of ecology. Plague is inherent within the natural plague focus and circulates among host and insect vectors in the environment where it seldom causes plague and is difficult to detect by regular surveillance during rest period. |
| Introduction | | |
| Background/rationale | 2 | Plague, a *Yersinia pestis* infection, is a fatal disease with tremendous transmission capacity. However, the mechanism of how the pathogen stays in a reservoir, circulates and then re-emerges is an enigma [1]. Plague in China is shown to be in 12 kinds of natural foci with a large scale geographic and complex structure [2]. Each plague focus has a unique ecological environment, specific geographic regions, landscape characteristics and specific hosts and vectors for the maintenance and transmission of *Y. pestis* [2-3]. The emergence of enzootic plague is a kind of natural reservation for the pathogen [4]. Humans are infected with disease via a route such as bite by the flea from infected rodent [5-7]. *Y. pestis* continuously circulates between a host-vector-environment complex where the interaction determines the prevalence of the pathogen [5, 8-10]. Once the environment alters, the amount and density of hosts and vectors change and so does the survival of *Y. pestis* [11-12]. Consequently, a rest or slightly active foci come alive, and rapidly causes plague among animals and or humans [13]. |
| Objectives | 3 | A plague outbreak among animals and humans caused by the construction of a large reservoir in southwest China is reported in this study. The reservoir was located on the border between Yunnan-Guizhou Plateau and the Guangxi Hills on the Nanpan River. The plague outbreak took place along the repository of the Tianshengqiao reservoir in the border region of three provinces in southwest China: Xingyi County and Anlong County of Guizhou Province, Longlin County and Xilin County of Guangxi Province, and Luoping County of Yunnan Province. Since the outbreak, the region was identified as the Rattus flavipectus plague foci belonging to the mountainous area of Western Yunnan and the coastal area of Fujian and Guangxi.  During the 10 years of reservoir construction, population density surged because construction workers relocated here. However, no enzootic or human plague was reported or infected dead rats found. When the impoundment inundation started in 1997, dead rats were present along the reservoir banks, and then by a few village banks in 1998. In 1999, the water capacity and drowned area increased, and the dead rat shot up and spread upstream from the reservoir banks to new branches. In 2000, dead rats were found in local farm houses in 15 foci along the banks (Fig. S1). In July of 2000, human plague occurred and it gradually declined until 2003 when no further cases were reported. An epidemiology study showed before the onset of plague, all the patients had dead rat contact and flea bite history. This outbreak of bubonic plague was transmitted by flea vector (Xenopsylla cheopis) and circulated between rat-flea- human. |
| Methods | | |
| Study design | 4 | Routine surveillance since the outbreak and the Control Surveillance |
| Setting | 5 | Immediately after the outbreak and for 16 years plague surveillance was performed in Xingyi County of Guizhou Province, Longlin County and Xilin County of Guangxi Province and Luoping County of Yunnan Province. Enzootic plague was monitored at both fixed and mobile surveillance sites. Rodent hosts were measured for population structure, density and seasonal change; and importantly were examined for pathogen and serum detection of antibody and Y. pestis antigen. Human case reports were collected.  The natural plague foci in Gansu Province are relatively active, some of which are long prevalent, and some often alter between active and rest periods. We analyzed the surveillance and epidemiology data of some natural plague foci in Gansu from 1959 to 2014 and set the long active the Himalayan marmot foci of the Qilian-Altun Mountains as study control. |
| Participants | 6 | Rodent hosts, indicator animals and flea vectors  The cage-trap method was applied for estimation of rodent density (the number captured/the number of the rat cages) in the plague foci from surrounding farmhouses. Self-dead rodents from monitoring areas were collected. All the rats were classified and identified for population composition and the density was calculated with the captured rodents. Fleas carried by the rats were collected and counted; identification was performed under optical microscopy. They were pooled for Y. pestis culture. Dogs and cats infected Y. pestis by catching and feeding on infected rats, they have mild symptoms and are often self-healing, but specific F1-Antibody can be shown positive in the serum [14-16]. Dogs and cats are believed to be a surveillance sentinel by measuring the antibody to F1 in the serum to predict plague outbreak risk [17-18]. Based on rodent surveillance, pathogen and sera were monitored in indicator animals, dogs and cats, to indirectly assess plague prevalence and they were sampled every year. |
| Variables | 7 | Pathogen detection  Samples from all the live and died rodents (including blood, liver, spleen, lung) were obtained for inoculating onto 2 selective agar plates (termed BIN agar, which is based on brain heart infusion agar with adding the selective agents of irgasan and cholate salts) for bacterial isolation at 28°C [19]. Flea were milled and inoculated onto culture medium. Biochemical tests, such as arabinose, glycerine, rhamnose, and melibioseuse; Gram staining; bacteriophage lysis test; and specific polymerase chain reaction tests targeting F1 and pla genes, were used to identify the suspected isolates [19-20].  F1 Antibody and antigen detection  Reverse indirect hemagglutination assay (RIHA) was used to detect the F1 antigen of Y. pestis from the tissues of naturally dead rodents [21]. The live rodents’ serum samples were collected from the femoral artery and were taken to detect antibody against F1 antigen by indirect hemagglutination assay (IHA). IHA was performed by including F1 antigen inhibition control and negative and positive controls. Antibody titers ≥1:20 (micro-plate method) was identified as positive; and required confirming analysis using the tube method according to the diagnosis criteria for plague [17, 22-25]. Captured rats that died of injury were not available for blood samples. Dogs and cats raised in Longlin County were tested for the F1 Antibody; they were calmed down and taken care of after venous blood collection; no pet animals died. |
| Data sources/ measurement | 8* | not applicable |
| Bias | 9 | not applicable |
| Study size | 10 | not applicable |
| Quantitative variables | 11 | not applicable |
| Statistical methods | 12 | Descriptive study. Not applicable. |

Continued on next page

| Results | | |
| --- | --- | --- |
| Participants | 13* | not applicable |
| Descriptive data | 14* | not applicable |
| Outcome data | 15* | not applicable |
| Main results | 16 | **Information on the plague outbreak**  A total of 210 human cases confirmed as bubonic plague with only one dead with exacerbation to sepsis. Clinical specimens from patients including the bubo aspirates, bloody sputum, throat swabs, and necropsy organs (liver, lungs, spleen and heart) were used to isolate and identificate of *Y. pestis*. In 1999, plague first appeared in Xingyi County, Guizhou Province. Seventeen plague patients were first diagnosed as acute lymphadenitis. The IHA test results of them showed a high titer of specific antibody for *Y. pestis* (titer≥1:80); therefore these 17 patients were retrospectively confirmed as having plague infection.  The human plague outbreak peaked in 2000, the first site of the outbreak, Xingyi county, was most serious during this year. The circulation of plague extended from the core region to upstream in the reservoir and its branches to the center of Xingyi and to the county nearby the reservoir, Anlong and Longlin. Eighty-eight human plague cases were from Xingyi county, 44 from Bajie and 28 from Jushan, which were the most severe sites. Tianshengqiao in Longlin County, where the reservoir was, also experienced a serious human plague with 40 patients spread (Fig. 1).  In 2001, with the adoption of prevention and control measures the plague extend from the core focus to upstream and to branches of the reservoir, with Xingyi (24 cases), Longlin (9 cases) and Xilin (5 cases).  In 2002, due to effective control measures, plague rapidly weakened. Jushan, at the end of a branch had the most patients (10 cases), while Nidang with six cases, Xiawutun with five cases, Bajie with two cases, and Zerong with one case (Table 1).  In 2003, only one case appeared at Bajie in the core focus, Xingyi (Table 1 and Fig. 1), and was the last case. The index and the last case both emerged in the most seriously impacted county, Xingyi.  Regional distribution of foci and spread path of plague  Before the water impoundment of the reservoir, a large number of residents lived along the banks of Nanpan River with large quantities of farmland. After filling, the river was wider, and the capacity increased; and as a consequence the farmland along the banks was submerged, covering a total area of 177.75 square kilometers; and brought about 42,050 immigrants (Fig. S1-A, B, D). Human plague started in the villages along river banks of Tianshengqiao Reservoir (Tianshengqiao, Wanfenghu, Bajie, Gebu were the core foci of the outbreak), and gradually spread upstream and to the branches of the reservoir. Most foci were distributed in villages along the banks and branches of the reservoir. Among them, Tianshengqiao, Wanfenghu, Bajie, Gebu, Zerong, Xiawutun, Jushan, Dingxiao, Nidang, and Bada were the sites where both human plague and enzootic plague occurred. In Jinzhongshan, Cangjiang, Luowan Sanjiangkou, and Lubuge, only enzootic plague took place and no human cases were reported; these places belonged to the subsequent dissemination region (Fig. 1, S1C). Dead rodents, from which Y.pestis were isolated, were found in farmhouses in Luoping, Yunnan Province located upstream of the reservoir and these farmhouses were only 6 kilometers away from the bank of the tailrace submerged by the reservoir. Further, a number of natural dead rodents were found in the field beside the farmhouses (Fig S1-E).  **Routine surveillance**  The rodent hosts and indicator animals  Surveillance of rodent density after the outbreak  A small amount of dead rats were found along the reservoir bank in 1998. The dead rats increased in 1999 with the impoundment where the submerged area increased. By 2000, dead rats were found not only along the reservoir banks, but also in nearby resident houses along the riverside. The plague was prevalent among the rodents after with a large number of self-dead rodents were found in the epidemic area. The human plague began in July of 2000, and prevalent in areas where the dead rats increased with the water impoundment processes, no dead rats no plague cases.  The surveillance was conducted in Longlin, Xilin, Luoping and Xingyi Counties from 2000 to 2014. The average rodent capture rate from the four counties was 3.77% (30,524 /809,652). Total capture rate from low to high were respectively Xingyi 3.04% (6,751/221,820), Longlin 3.06% (7,796/254,655), Xilin 3.94% (8,855/224,850), and Luoping 6.57% (7,122/108,327). From 2000 to 2001 after the outbreak, Xingyi enforced de-ratting and thus failed to carry out normal rodent density surveillance. With transverse comparison of the rodent capture rate in 4 counties in 2002 and 2003, Longlin was 3.20% and 2.78%, Xingyi 6.26% and 4.49%, Xilin 11.50% and 5.26%, and Luoping 8.62% and 12.25%, respectively. The rodent composition indicated R. flavipectus was the dominant host in Xingyi, Xilin, and Longlin, while it was R. norvegicus in Luoping. We found respectively:  Xingyi: R. flavipectus (41.1%) > R. norvegicus (38.0%) > M. musculus (11.7%);  Xingyi: R. flavipectus (85.3%) > M. musculus (10.0%) > R. norvegicus (11.7%);  Longlin: R. flavipectus (82.5%) > M. musculus (11.0%) > R. rattus sladeni (2.3%);  Luoping: R. norvegicus (57.4%) > R. flavipectus (32.5%) > M. musculus (8.0%).  *Y. pestis* isolation from rodents  Tissues from a total of 39,166 rodents were cultured from the four counties from 2000 to 2014. Y. pestis strains were only isolated from 2000, 2001 and 2002 consistent with the emergence of human cases. A total of 64 strains were isolated where the average carriage rate was 0.16%; and respectively from high to low were Xingyi 0.21% (27/12,680), Longlin 0.16% (13/7,945), Xilin 0.16% (18/10,912), and Luoping 0.08% (6/7,629). Specifically, in Xingyi we isolated strains from 2000 to 2002: 5.45%, 7.69% and 1.55%. And from XiLin we isolated Y pestis in 2001 and 2002, 1.68% and 0.31% respectively. From Longlin, we only isolated strains in 2000 (3.82%). From Luoping strains were isolated in 2001 (0.92%) (Fig 2, Table 2). The results fully confirm R. flavipectus as the dominant rodent host in the plague foci. Host density investigations show R. flavipectus is the highest in all of the counties except Luoping, where R. norvegicus is slightly higher than R. flavipectus. However, Y. pestis strains are predominantly isolated from R. flavipectus in all counties.  The detection of F1 antibody  A total of 22,472 rodent serum samples were collected from the four counties during 2000-2014 and F1 antibody tests were performed using the indirect hemagglutination test (IHA). Twelve were positive, and the average positive rate was 0.053%. We collected 6,752 serum samples from Xingyi during 2002-2014 (routine surveillance was not performed from 2000 to 2001 due to the de-ratting operation), and five were positive with positive rates were respectively 1.58% (3/190) in 2002, 0.14% (1/700) in 2003, and 0.2% (1/500) in 2006. Among 4,810 samples collected in Longlin, six were positive (all R. flavipectus). Among 8,543 samples collected in Xilin, only one R. flavipectus was positive in 2001, and its positive rate was 0.15% (1/674). All 2,367 serum samples collected in Luoping during 2000-2014 were IHA negative (Fig 2, Table 3). IHA positive results primarily came from R. flavipectus, consistent with the strain isolation results. Besides positive samples detected during the plague epidemics, one positive serum came from R. flavipectus in Xingyi in the rest phase (in 2006).  F1 antigen data from liver and spleen samples from naturally dead rodents  Liver and spleen samples, 1,047 in total, collected from naturally dead rodents in the four counties during 2000-2014, were detected using the reverse indirect hemagglutination test (RIHA). The average positive rate was 25.21%, and positive specimens primarily appeared in 2000-2003; and generally in accordance with the emergence of the human cases. Positive rates from high to low were respectively Xingyi 45.77% (195/426), Luoping 38.89% (21/54), Longlin 16.13% (25/155), and Xilin 5.58% (23/412). The positive results in Xingyi County gathered in 2000-2003 were respectively, 49.82%, 45.76%, 71.79%, and 15.00%, while the rest of the years are negative. The positive samples of Longlin gathered in 2000-2002 were, respectively, 35.71%, 25.49% and 33.33%, while the rest negative. Xilin was 36.73% and 9.30% in 2001 and 2002 respectively, and in 2010, during the rest period, one R. flavipectus sample tested positive. The RIHA test in Luoping County was performed only in 2001, and its positive rate was 38.89% (Fig. 2).  Surveillance of indicator animals (high-resistant animals)  Dogs and cats are of important indicator animals to predict the epidemic intensity of plague. Both China and other countries have related reports that these two indicator animals have low sensitivity and a high tolerance for Y. pestis [15-17]. The serological surveillance number of indicator animals between plague foci and non-plague foci in Longlin County from 2000 to 2014 were 2,113 and 1,592 respectively, the result was as follows: in the plague foci, the IHA positive rate from cats during 2000–2002 were 30.95%, 13.33% and 33.33% respectively, and in the rest of the years were negative; the IHA positive rate from dogs from 2000, 2002 and 2003 were 11.85%, 3.66% and 0.91%, respectively, and the rest of the years negative. In contrast, the results from dogs and cats in non-plague foci were all negative. Cats have a special addiction to small rodents, thus the cats' positive rate was higher than farm dogs (Fig 3).  Flea vector surveillance:  During 2000-2014, 15,696 vectors were cultured for Y. pestis isolation in the four counties, among which only Xingyi County, the severest human plague foci, had isolated strains. The average positive rate of Xingyi was 0.47% (12/2,537), specifically 30.43% (7/23) in 2000 which were all isolated from X. cheopis; 21.05% (4/19) in 2001, one from X. cheopis and three from M. anisus; 1.10% (1/91) in 2002 isolated from X. cheopis. The time distribution of positive vectors was coincident with human case appearance. In addition, 6,019 vectors were collected in Longlin, 3,898 in Luoping, and 2,537 in Xilin, all of which were negative for Y. pestis strains (Fig. 2).  We collected the rodents including live rats and dead rat, plague bacteria could be separated from dead rat, and mostly isolated from dead rat, and however serum samples of dead rat were not collected. Therefore, the IHA test can’t be performed. The plague bacteria were isolated from the majority of IHA positive rodents in our study. Some serum samples were positive for IHA test, while couldn’t isolated the bacteria. Meanwhile, flea and rats did not have a corresponding detection, we can’t know whether the test positive rat fleas from the positive rodents.  **Surveillance for control foci**  The epidemic situation in a long-active natural plague foci  In Gansu Province from 1959 to 2014, outbreaks of human plague happened 30 times, in which 70 patients were diagnosed; and 43 of them died with a fatality rate of 61.43%. Between 2001 and 2014, the Himalayan marmot foci of Qilian Mountain was particularly active, with one human case in 2004, two cases in 2007, one case in 2010, and three cases in 2014. This focus belongs to the China Himalayan marmot foci on the Qinghai-Tibet Plateau, where the plague is enzootic among animals. The surveillance results during 2001-2014 showed that the isolation rate of host was 1.83%; the isolation rate of vectors was 1.09%; the average IHA positive rate was 3.03%; and the average RIHA positive rate was 4.35% (Fig. 4).  An epidemic in a natural foci turning into the rest phase after an active phase  The Gannan Tibetan Autonomous Prefecture is another natural focus in Gansu Province, located on the Marmatahimalayana plague focus of Qinghai-Tibet plateau. The first human plague broke out in 1958, and later the plague was enzootic between animals in the local grassland habitat of Marmatahimalayana until 1970. During 1971 to 1990, no plague was detected in this region. But in 1991, two marmots were detected F1 Ab positive, and then five in 1998, two in 1999, and one in 2000; the highest titer was 1:160. In 1999 a large number of marmots died. Three strains of Y. pestis were isolated from Marmatahimalayana, and eight samples were positive for RIHA (five at 1:100, and three at 1:200).  The third plague natural foci in Gansu Province is the plague focus of Spermophilus alaschanicus Buchner on the Loess Plateau in Longzhong, belonging to the plague foci of Spermophilus alaschanicus Buchner in the hilly grassland on the Loess Plateau of Gansu and Ningxia. After a human plague outbreak in 1962, animal plague was enzootic until 1977. And from 1977 to 2014, only one animal was positive for the F1 antibody (1:80 in 1977), and no enzootic plague was found. |
| Other analyses | 17 | not applicable |
| Discussion | | |
| Key results | 18 | Plague has a strict geographical distribution, which is called plague natural foci. Plague invades, fades out, and re-invades alternately existing in animals where some plague natural foci seem to disappear permanently or re-invades after a rest period of many years. The reasons for plague's persistence, abrupt disappearance in natural environment are poorly understood [27].  Here we report an outbreak of plague occurring on the border between Guizhou, Guangxi and Yunnan resulting from the building of a large reservoir. The last disease related to the death in rats, based on historical records in Luoping County, Yunnan Province, occurred from 1882 to 1892. The pandemic spread over eleven years, leaving a human death toll of ten thousand. Although it has not been confirmed today, we speculated it was a plague through the description of the event and the characteristics of the outbreak in this study. Since then no similar incident has been reported in the area for a hundred years. Until 1997, the animal and human plague re-invaded after the impoundment of reservoir in the late 1990s. The plague lasted until 2004, when human plague disappeared and animal plague faded out with only a few rodents carrying the F1 antibody or antigen. We speculate the valley region along the Nanpan River may be a natural plague focus previously in history where under certain conditions, the plague invaded and faded out into a long rest period. The Y. pestis bacteria circulated in a very limited range and formed several micro-foci with discrete distribution and were hard to discover as the infection was in a long resting stage. When the dam began to fill the valley, the ecological environment experienced a violent change in a short period of time with the water level increasing. Animal migration resulted in the population density and a distribution change in rodent hosts. The plague-infected rodents previously in scattered distributed micro-focus gathered; then, the plague re-invaded. When the animals adapted to the new habitats and recovered to a stable state, the plague foci returned to the resting stage. This highly supports the inner preservation theory of plague [20], which states Y. pestis exist within plague foci in the resting stage; however it is weakly prevalent and difficult to detect.  During the ten years constructing the reservoir, there were no epidemic plague among human and animals. A small amount of dead rats were found along the reservoir bank in 1998. The dead rats increased in 1999 with the impoundment where the submerged area increased. By 2000, dead rats were found not only along the reservoir banks, but also in nearby resident houses along the riverside. The plague was prevalent among the rodents after with a large number of self-dead rodents were found in the epidemic area. The human plague began in July of 2000, and prevalent in areas where the dead rats increased with the water impoundment processes, no dead rats no plague cases. Plague initially existed in the first drown areas by the impoundment of the reservoir (Fig. 1, the red zone ), foci were mainly distributed in the submerged regions along the riverside and its branches, later spreading towards the upstream and to branches of the reservoir (Fig. 1 and S1 show the transmission route).  Enzootic and human plague has persisted until 2003, although the initial confirmed case was in 2000, the first cases can be traced back to 1999 when 25 patients had acute lymphadenitis according to the local hospital records. After the outbreak in 2000, they were tested with IHA, and 17 of 25 were detected positive with F1 antibodies (with titers>1:80), which were then diagnosed as plague cases. This fully confirms plague cases occurred in 1999 without being discovered, and the dead rodents existing in the early stage of impoundment were associated with the plague infection. A total of 210 human cases occurred in this outbreak and were all confirmed as bubonic plague with only one dead because the patient delayed in seeing a doctor with exacerbation to sepsis.  Plague spread requires both a high abundance of hosts and a sufficient number of active fleas. The surveillance results show the plague epidemic was worst in Xingyi County, however the rodents captured by cage traps was lower than Xilin and Luoping County, only higher than Longlin County, which may have been due to the de-ratting before the monitoring and a large number of rodent death during the plague outbreak. However, the Y. pestis isolation rate in rodents in Xingyi was much higher than the other three counties from 2000 to 2002. The flea was the primary transmitting vector for plague where its populations and bacteria carrying rate directly related to the outbreak and prevalence of plague [7, 28]. In this study, Y. pestis was only detected from the fleas in the core outbreak zone, Xingyi County, and the bacteria isolation rate from fleas was in accordance with the number of plague cases among people from 2000 to 2002. After the plague epidemic (after 2003), plague retreated and the natural foci seemed to rest for a long time, and it was difficult to detect in animals in routine surveillance from 2004 to 2014 (Fig. 2). Only one IHA positive R. flavipectus was discovered in Xingyi County in 2006 (positive rate 0.2%), and one RIHA positive naturally dead R. flavipectus in Xilin County in 2010 (positive rate 1.08%). Thus, the plague has retreated over the past decade, but it has not disappeared. Due to the limited number of naturally dying animals available during the conventional monitoring, it was difficult to detect the extremely low levels of plague prevalence in the resting stage. To find a better way of monitoring, it was necessary to strengthen the routine surveillance. Accordingly an additional enhanced surveillance was performed in the four natural foci (counties) during 2013-2015, two canines and one R. flavipectus were examined positive for F1 antibody in Xingyi County in 2014, and in Luoping in 2015, respectively. This was further evidence Y. pestis still exist and is multiplying in the rodent hosts in the resting natural plague foci.  Though cats could be infected by Y. pestis, they are believed to be a surveillance sentinel by measuring the antibody to F1 in the serum to predict plague outbreak risk [17-18]. The cats can resistant to Y. Pestis infection [16] and was a mediator to facilitate transfer of fleas as a source of bubonic or septicemic plague [29]. As we all know China is one of the most prevalent countries of plague, and therefore the responsibility of the government was to define each plague natural focus so the people can understand the major characteristics of plague and be aware of its dangers. However, the region in this study was not defined as a potential plague natural focus before 2000, so the government did not pay close attention and take emergency measures until the outbreak of plague cases. It suggested that timely recognition and judgment of the natural foci of plague is vital in the treatment and prevention of this acute and highly infectious disease. It was easy to overlook the dangers of plague especially for the resting plague natural foci where it is difficult to discover animal plague at a low epidemic stage. The plague will re-invade when the environment or climate change, and threats of outbreaks may thus be increased where local humans live in close contact with rodents and fleas (or other wildlife) harboring endemic plague.  Emergence, spread, persistence and fade-out of plague can alternate for varying lengths of time in the plague natural foci [13], the reasons for plague’s persistence and abrupt disappearance are poorly understood. We speculate the plague occurs in a particular habitat, the micro-focus, and Y. pestis is preserved in this region during the resting stage. Similar to our study, the human plague broke out in Spermophilus dauricus alashanicus plague natural foci in Huining County, Gansu Province in 1962, and a large number of rodents died before the epidemic. The enzootic plague lasted until 1977, since then no Y. pestis strain isolated, with only one Spermophilus dauricus alashanicus detected positive with F1 antibody in 1997. In the Gannan Tibetan Autonomous Prefecture, Gansu Province, located on the Qinghai-Tibet plateau plague focus of Marmatahimalayana, human plague started in 1958 and the epizootic plague persisted among marmot until 1970. After a resting stage from 1971 to 1990, Y. pestis strains were isolated from marmots again from 1991 to 2000; and thereafter the plague faded-out in this region from 2001 until now. The Qilian Mountain region of Gansu Province which is also located on the Qinghai-Tibet plateau plague focus of Marmatahimalayana is one of the most active natural plague foci in China [15, 30], witnessed by continued outbreaks of plague in animals year after year with Y. pestis persistently isolated from marmots (except from 1979 to 1981); especially in 2014, three human plague patients were found within three months. However along the Nanpan river, there has a large population in the reservoir area, after constructed the reservoir, the local people make a huge migration, and the changed the landscape destroied the suitable environment for rodents. As a result, the rodent densities were decreased, and the plague prevalence faded.  There is a distinct difference in the ecology environment, human population and animal density in these three areas. Huining County with a high concentration of people (580,200 people on 5,657 km2). After the outbreak of plague in the 1960s, the local people cultivated the virgin land, changed the landscape to destroy the suitable environment for Spermophilus dauricus alashanicus. As a result, the rodent densities were decreased, and the plague prevalence faded. The Gannan Tibetan Autonomous Prefecture was similar with a large population and tourism development, so the active and rest period of plague alternate in this region. However, sparsely populated in Subei (12,000 people on 55,264 km2) and Akesai County (8,000 people on 29,142 km2) in the Qilianshan region were beneficial to the persistence of plague. Fewer construction projects and dispersed residences were also conducive to keeping the ecological balance of plague, with sporadic or only local breakouts (Fig. 4) and few human cases occurred. It is common for re-emerging rodent-derived plague epidemics after decades in many regions of the world [11, 13]. For instance, in Kazakhstan, the bacterium Y. pestis circulates in natural populations of gerbils. Analysis of field data collected between 1955 and 1996 shows that plague invades, fades out, and re-invades in response to fluctuations in the abundance of its main reservoir host, the great gerbil (Rhombomys opimus) [13].  The spread of plague requires interactions with the pathogen, its ecology, hosts and vectors [12-13, 31]. Here we show a typical example of the re-emergence of plague driven by the construction of a large reservoir. Through the comparison study of the Qinghai-Tibet plateau plague focus of Marmatahimalayana in Gansu province, we confirmed that the bacterium Y. pestis can long-term persist in the natural plague focus [27, 32] where it exists within the micro-focus in the rest stage, and persists covertly among its hosts and vectors. When the host density increases or abiotic factors change, a local outbreak of plague occurs. The inner preservation theory of plague is supported by this outbreak and the subsequent 16-year continuous monitoring, states that Y. pestis is inherent within a natural plague focus. |
| Limitations | 19 | not applicable |
| Interpretation | 20 | not applicable |
| Generalisability | 21 | not applicable |
| Other information | | |
| Funding | 22 | This work was supported by the National Natural Science Foundation of China  (General Project, no. 81470092) and the National Sci-Tech Key Project  (2012ZX10004201, 2013ZX10004203-002). |

*Give information separately for cases and controls in case-control studies and, if applicable, for exposed and unexposed groups in cohort and cross-sectional studies.

**Note:** An Explanation and Elaboration article discusses each checklist item and gives methodological background and published examples of transparent reporting. The STROBE checklist is best used in conjunction with this article (freely available on the Web sites of PLoS Medicine at http://www.plosmedicine.org/, Annals of Internal Medicine at http://www.annals.org/, and Epidemiology at http://www.epidem.com/). Information on the STROBE Initiative is available at www.strobe-statement.org.
