# Supplementary figures and images for: Mechanism study on a plague outbreak driven by the construction of a large reservoir in southwest china (surveillance from 2000-2015)
Source: PLoS Negl Trop Dis. 2017 Mar 3;11(3):e0005425. doi: 10.1371/journal.pntd.0005425 (PMC5352140; doi:10.1371/journal.pntd.0005425)

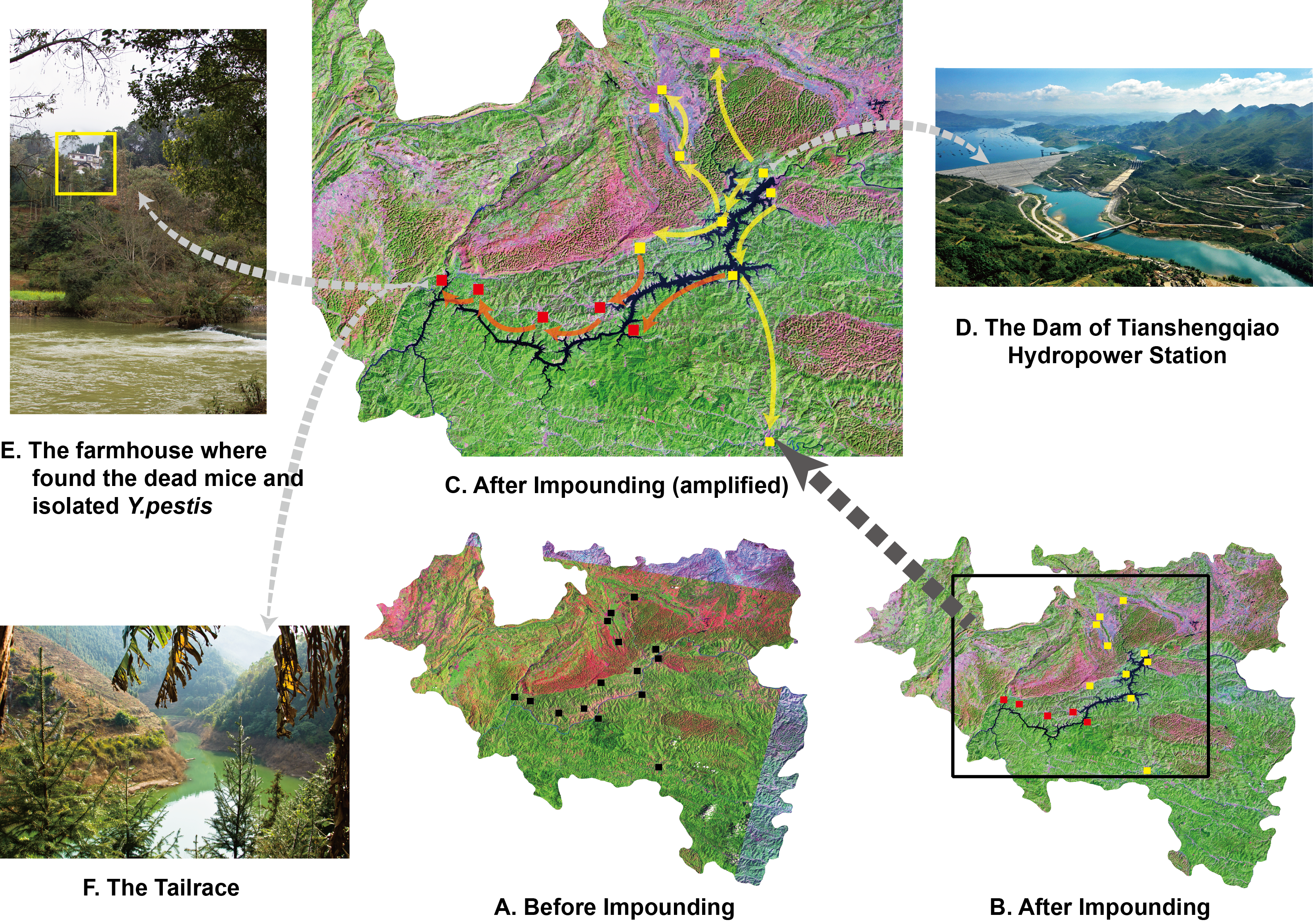

Supplement: S1 Fig — A: Landscape before the impoundment; the black dots represent foci with both enzootic and human plague cases; B: Landscape after the impoundment; the yellow dots represent foci with both enzootic and human plague cases; the red dots represent foci only with enzootic plague but no human plague; C: The enlarged view of black rectangle of B; the yellow dots represent the foci with both enzootic and human plague cases; yellow arrows represent the spread path of enzootic and human plague; the red dots represent the foci with only enzootic plague but no human plague; red arrows represent the subsequent spread path of enzootic plague. D: Aerial photo of the Tianshengqiao reservoir upstream and downstream. E: The farmhouse on the bank of the tailrace where dead rodents were found and Y. pestis strains were isolated; the yellow box indicated the farmhouses on the banks. F: Environment of tailrace banks. The map is a Topographic Database of the National Fundamental Geographic Information System of China (NFGIS) which is shared and for free use, the URL link was http://www.tianditu.com/. (TIF) [file pntd.0005425.s002.tif]
