# Supplementary material for: Mechanism study on a plague outbreak driven by the construction of a large reservoir in southwest china (surveillance from 2000-2015)
Source: PLoS Negl Trop Dis. 2017 Mar 3;11(3):e0005425. doi: 10.1371/journal.pntd.0005425 (PMC5352140; doi:10.1371/journal.pntd.0005425)
Supplement: S2 File — (PDF) [file pntd.0005425.s004.pdf]

中国疾病预防控制中心传染病预防控制所

伦理审查委员会批准通知书

NATIONAL INSTITUTE FOR COMMUNICABLE DISEASE CONTROL AND PREVENTION CHINESE CENTER FOR DISEASE CONTROL AND PREVENTION

ETHICAL COMMITTEE APPROVAL NOTICE

NO：ICDC-2015002

PRINCIPAL INVESTIGATOR OF PROJECT: Dr. WANG Xin

TITLE OF PROJECT: Pathogen Spectrum Study on Epidemic Regularity and Variability of Natural Foci Infectious Diseases

PROJECT DURATION: FROM Jan 1, 2012 TO Dec 31, 2015

DEPARTMENT/DIVISION: Laboratory of Emergency Response, IDCD

FUNDING AGENCY: Ministry of Science and Technology of the People's Republic of China funded programme: the National Key Science and Technology Project on Infectious Disease Surveillance Technique Platform of China (No. 2012ZX10004201)

DATE SUBMITTED: Feb 21, 2014

DATE APPROVED: Apr 21, 2014

The project entitled "Pathogen Spectrum Study on Epidemic Regularity and Variability of Natural Foci Infectious Diseases", submitted by investigator Dr. WANG Xin, Department of Laboratory of Emergency Response, has been approved by the meeting of ethics committee of national institute for communicable disease control and prevention, China CDC, according to Chinese ethics laws and regulations. It is recognized that the right and the welfare of the subject are adequately protected. The Investigator should submit summaries of investigation to the ethics committee annually.

SIGNATURE ____________

XU Jianguo

Chair, Ethical Committee

National Institute for communicable disease control and prevention

China CDC

DATE:
